# An overview of the oil-brine interfacial behavior and a new surface complexation model

**DOI:** 10.1038/s41598-019-42505-2

**Published:** 2019-04-15

**Authors:** María Bonto, Ali A. Eftekhari, Hamidreza M. Nick

**Affiliations:** Danish Hydrocarbon Research and Technology Centre, Technical University of Denmark, Elektrovej building 375, 2800 Kgs, Lyngby, Denmark

## Abstract

The few existing surface complexation models (SCM) for the brine-oil interface have important limitations: the chemistry of each crude oil is not considered, they cannot capture the water/non-polar hydrocarbons surface charge, the interactions between Na^+^ and the acid sites are not included, and the equilibrium constants for the adsorption reactions are not validated against experimental data. We address the aforementioned constraints by proposing an improved diffuse-layer SCM for the oil-brine interface. The new model accounts for the chemistry of crude oils by considering surface sites linearly dependent on the TAN (total acid number) and TBN (total base number). We define weak sites to account for the negative surface charge observed for non-polar hydrocarbons in water. We optimize the parameters of our model by fitting the model to reported zeta potential measurements of oil in aqueous solutions. When we validate the optimized model against different experimental data sets, it generally shows a good performance in predicting the surface charge of oil in different brines with different pHs. We show that the acid and base numbers are only useful as a qualitative estimation of the distribution of polar groups at the oil surface, and more sophisticated analysis is necessary to quantify the chemistry of the oil-brine interface.

## Introduction

The increased oil recovery during low salinity water flooding is a consequence of the crude oil/brine/mineral interactions. While attempting to get more insight into this recovery method, the majority of studies have focused on the brine/mineral interactions. However, the idea is recently gaining popularity that the increased oil recovery comes to a great extent from the fluid-fluid interactions^1–4^. Several mechanisms related to the oil/brine system have been reported as responsible for the increased oil recovery: wettability alteration^4,5^, viscoelasticity of the brine-oil interface^1,3,6,7^, interfacial tension (IFT) alteration^8^, emulsion formation^2,9^, and viscosity decrease^2^. All these mechanisms, except wettability alteration, are linked exclusively to the fluid-fluid interactions. In the following, we give an overview of the previous studies of the oil-brine interactions and their relevance to the mentioned mechanisms.

The wettability alteration relies upon the stability of the water film between the rock-brine and brine-oil interfaces, which depends on the development of surface charges at these interfaces. Comparable to metal oxide surfaces (e.g. calcite), the interface between oil and water becomes charged due to the occurrence of acid/base interactions and adsorption reactions^10^. The oppositely charged ions present in the brine are attracted to the water-oil interface, which increases their concentration at the interface and forms a diffuse ionic layer. The thickness of the diffuse layer is related to the Debye length and therefore to the ionic strength. At lower ionic strength, the polar components of the oil may migrate to and rearrange at the interface due to electrostatic attractions. However, when the salinity is increased, the Debye length is shorter, decreasing the attraction of the polar components^6^. The accumulation of active species at the interface will lower the IFT. The changes in the surface charge at the oil-water interface will also impact the interactions between oil droplets leading to changes in the rheological properties of the system^11,12^. The surface charge is indirectly estimated through electrophoretic mobility studies, which can be related to the zeta potential. The zeta potential measures the electrical potential at the surface of shear and indicates the adsorption and desorption of ions into the Stern layer^13^. With the goal of finding the optimum concentration for the injection brine during modified salinity water flooding, Jackson *et al*.^5^ proposed to use zeta potential measurements at both brine-oil and mineral-brine interfaces. They correlated the cumulative increase in oil recovery with the cumulative normalized zeta potential, showing that a higher cumulative zeta potential leads to increased oil recovery. Through their integrated crude oil/brine/mineral zeta potential measurement, they showed that the oil-brine interface could be positively charged. Therefore, in such cases, the injected water composition should be selected in a way that also yields a positive zeta potential at the brine/mineral interface. Identical zeta potential polarity at both interfaces leads to repulsion and improves the stability of the water film, altering the wettability towards a more water-wet state. Sari *et al*.^14^ also highlighted the importance of the zeta potential at both oil/brine and mineral/brine interfaces, and furthermore found a correlation between the absolute value of the sum of the zeta potential at the two interfaces and the contact angle, whereby a higher modulus (i.e. identical polarity at the interfaces) corresponded to a lower contact angles (a more water-wet state). In the same way, Xie *et al*.^15^ suggested studying the charges at both interfaces, since the double layer expansion would be responsible for the wettability alteration. Alshakhs *et al*.^4^ carried out zeta potential measurements at the rock/brine and brine/oil interfaces to discern which interface has a higher impact on the contact angle and disjoining pressure. They concluded that the wettability alteration is caused mostly by the brine/oil interactions. On the contrary, Lu *et al*.^16^ showed more skepticism regarding the relationship between the zeta potential and the contact angle. They explained that zeta potential measurements do not reflect the properties of the thin film interface, but rather the features of a region further away from this film. Therefore, the role of the thin water film properties on the wettability alteration might not be truly taken into account when a contact angle is inferred from these measurements.

The DLVO (Derjaguin-Landau-Verwey-Overbeek) theory has also been widely used to explain the stability of emulsions, which are highly unstable colloidal systems. Higher electrostatic repulsive forces and therefore higher zeta potentials theoretically increase the stability of these systems, while higher attractive van der Waals forces lead to instability^13^. García Olvera *et al*.^1^ studied the changes in the viscoelasticity (a rheological property) and the emulsion stability of a sulfate containing brine and different crude oils. A relationship between the asphaltene content and properties of the interface was observed: crude oils with higher asphaltene content showed higher elastic and viscous moduli and an increased IFT. The IFT decreased when sulfate was added to the brine. Additionally, they reported that naphtenic acids showed the opposite effect, destabilizing the emulsions. Contrary to this, Moradi *et al*.^17^ found that both asphaltenes and naphtenic acids improve emulsion stability. They revealed that a higher ionic strength induces a better partitioning of the acids but inhibits asphaltene accumulation at the interface. Alvarado *et al*.^3^ reported that the improved oil recovery is due to a combination of alteration of rock wettability and the development of interfacial viscoelasticity. Snap-off, which consists of the separation of the oil phase into a droplet or oil ganglion, might occur during secondary waterflooding. If an elastic interface is built up, the snap-off phenomenon can be reduced, which alters the residual oil saturation and possibly the relative permeabilities; this consequently improves the oil recovery. In cases where the separation of the oil phase still occurs, some pore throats could be blocked due to the greater size of the droplets, caused by the lower ionic strength; this diverts the flow towards unswept zones. They attributed the observed oscillations in the pressure drop during a tertiary water flood to this phenomenon. They also observed a higher oil recovery at low salinity compared to high salinity water flooding, explaining it by the higher elasticity between brine-crude oil in the presence of low salinity water. Chavez *et al*.^6^ affirmed that injecting low salinity water allows the accumulation of amphiphilic components (i.e., components with both hydrophilic and hydrophobic parts) at the oil-brine interface, which increases the interface viscoelasticity, and suggest that this is the reason for the increase in the oil recovery. The higher interfacial elasticity prevents/reduces snap-off of the oil into small droplets, which leads to a more continuous interface that is easier to mobilize during water flooding. By measuring elastic and viscous moduli of the interface, they studied the change in the viscoelasticity as a function of salinity, cation type and the additional effect of a surfactant. They observed that the variation of viscoelasticity with salinity is nonmonotonic. A maximum in the viscoelasticity is observed at a specific salt concentration, beyond which the viscoelasticity decreases with increasing salt concentration. On the other hand, Ayirala *et al*.^18^ reported that, even though the connectivity of the oil phase increases with increasing viscoelasticity of the interfacial film, very high viscoelasticity does not necessarily imply a higher oil recovery. Instead, they considered the coalescence time to contribute more to oil connectivity, since this factor indicates the time that takes for the snapped-off droplets to reunite. In the interfacial shear rheology experiments, the highest viscous and elastic moduli were obtained for the sulfate-containing brine. However, higher coalescence times were also obtained with this brine, indicating more isolation between the droplets. Thus, contrary to other works^3,6^, the authors conclude that ions that give rise to less rigid films and promote faster coalescence, reduce oil snap-off, and increase the oil mobilization during water flooding. All these seemingly contradictory observations can be better explained by a mechanistic model that describes the physicochemical interactions at the crude oil-brine interface and sheds light on the consistency of the reported data.

The role of IFT in low salinity water flooding is a controversial topic. There is evidence that points to an increased oil recovery during low salinity water flooding due to a decrease in the IFT^8^. However, the opposite is reported in other works, which show a lower oil recovery factor due to IFT increase at lower salinities^19^. Lashkarbolooki *et al*.^20^ explain that the inconsistencies in the crude oil-brine IFT at different salinities arise from the different endogenous oil surface active components present in the different oil samples; this requires further investigation. Zahid *et al*.^2^ studied the formation of emulsions between three different crude oils and seven brines. They observed emulsification between aqueous solutions and oil at room temperature, especially in cases where distilled water or brine saturated in Mg^2+^ ions were used in the experiments. Although the ionic composition had an impact on the emulsification process, the authors could not establish any relationship between the salinity and the specific ion effect on the emulsion formation. Additionally, for one of the crude oils, at high temperature and pressure, they identified the formation of a possible microemulsion phase with increasing sulfate concentration, which could greatly influence the oil recovery. The brine composition was also found to affect the crude oil viscosity, with the highest viscosity reduction caused by sulfate ions, which were believed to promote a reorganization of the heavy components leading to a change in shape (“coiling”). Gachuz-Muro *et al*.^21^ also observed changes in the viscosity when putting different crude oils in contact with brine. They observed more alteration in high viscosity oils. Chakravarti *et al*.^9^ studied the effect of cation and anion types on emulsion formation between crude oil and brine. Emulsion formation was observed in all cases, and they inferred that the main contribution to emulsification was from heavier alkanes and acids. Therefore emulsion formation depends on oil composition (especially the content of polar components) and salinity, reaching a maximum at a specific salt concentration. Sulfate and phosphate were identified as the most effective anions in promoting emulsification, while calcium and magnesium were the most effective cations. Perles *et al*.^22^ reported that emulsions are stabilized through asphaltene adsorption at the oil/water interface and complexation of acidic groups with cations in the brine. They identified two basic steps in the stabilization process: first, accumulation of asphaltenes and resins at the oil/water interface and second, a restructuring of the molecules at the interface, maximizing the intermolecular forces, through an “enthalpy-driven process”^23^. Although thicker and more rigid interfacial films were formed with saline solutions compared to distilled water, they found that there was a specific salt concentration that gave the emulsion maximum stability. A higher salinity impedes the stabilization mechanism because of an excess of adsorbed molecules at the interface, which might result in greater compression of the interfacial film; additionally, repulsion between adsorbed molecules can destroy the interfacial film, destabilizing the emulsions. The stability of emulsions is therefore directly related to the adsorption energy of the interfacially active molecules of the crude oil/water interface: increased energy of adsorption at the interface can enlarge the thickness of the interfacial film, which decreases the distance between the droplets and contributes to the steric stabilization of the emulsions. Additionally, in a subsequent work^23^, they highlighted the importance of temperature and aging time on the rheological properties of the crude oil/water interface, since these factors affect the diffusion of the surface-active molecules through the bulk phase and their reorganization at the interface. They also carried out interfacial rheological studies and measured higher elastic moduli (i.e. recoverable energy stored in the interface) and viscous moduli (i.e. dissipation of energy) in brine systems compared to deionized water. They indicated that the interfacial film provides more resistance to deformation and coalescence, which stabilizes the emulsions.

This work focuses on the interactions that occur at the oil-water interface, trying to find a mechanistic model that describes the physicochemical interactions at the crude oil-brine interface. This model can serve as a foundation for explaining the role of oil and brine composition and their interactions on the interfacial properties of crude oil-brine systems, with several implications for the production and processing of crude oil. We first give a short overview of the existing models used to describe the electric properties of the oil/water interface. Then we modify one of the models by including additional adsorption reactions and considering new types of surface sites. Finally, we test the accuracy of the optimized model by comparing its results with different zeta potential measurements available in the literature.

## Previous Models for the Assessment of the Surface Charge at the Oil-Brine Interface

Chow *et al*.^24^ initially used the Ionizable Surface-Group model to predict the zeta potential of bitumen in brine solutions. This model assumes that the charge at the surface of bitumen comes from the dissociation of acid groups, which depends on pH and electrolyte concentration. To enable the prediction of the zeta potential using this method, the site density and the pK_a_ for the acid are required. These were calculated by fitting the model to electrophoretic mobility measurements. In a later study^25^, they also used this model to determine the surface charge of crude oil. Later, Buckley *et al*.^26^ observed a positive charge on crude oil at low pH. They extended the Ionizable Surface-Group Model to account for both acid and basic groups. All these studies assume that the zeta potential can be calculated as the potential at an unknown but relatively short distance from the onset of the diffuse layer, taken as 0.5 or 0.6 nm. They obtained a different combination of parameters as a function of the slip plane distance. However, since the number of sites is part of the optimization process, no clear correlation is established between the content of active components in the crude oil and the surface site density. Therefore this method would require an optimization procedure for each type of oil.

Das *et al*.^27^ propose a similar model for calculating the zeta potential of asphaltene in aqueous solutions. Their model considers the carboxylic and hydroxyl ionizable sites as proposed by Szymula *et al*.^28^. This model assumes that the zeta potential is equal to the surface potential. However, this method was not directly applied to oil-brine systems; they argued that crude oil might contain additional interfacially active material besides asphaltenes, which explains the lower zeta potential magnitudes for asphaltenes compared to crude oil.

Brady *et al*.^29^ proposed a surface complexation model to predict the zeta potential at the oil surface. They considered two surface sites: amine base sites and carboxylic acid sites. The basic sites only undergo protonation, yielding positive surface charges at low pH. The acid sites can undergo dissociation and also react with divalent cations in the brine. No interaction with monovalent ions is considered. Furthermore, they assumed an equal number of basic and acid sites. This model was also used in later works to reflect the chemical speciation with pH at the oil surface^30,31^ and to show a correlation between the number of bonds between charged species on the oil and rock surfaces, and the contact angle^31^. However, the actual capabilities of the model to fit experimental measurements of zeta potential were not demonstrated.

Qiao *et al*.^32,33^ also proposed a diffuse double layer model for the oil-brine interface. However, they did not account for the differences in the chemistry of different crude oils, and they assumed a constant number of carboxylic sites of 6 µmol/m^2^. However, the performance of their surface complexation model was not tested against any experimental data. They reported that their equilibrium constants were taken from Brady and Krumanshl^34^; however, the numbers reported in their manuscript differ from the values obtained by Brady and Krumanshl^34^.

A common shortcoming of all these models is their inability to reproduce the zeta potentials measured for hydrocarbons containing no polar (ionizable) components (neither amine nor acid sites). Several works^35–38^ reported very negative zeta potentials for non-polar hydrocarbons (with no ionizable surface sites) in aqueous solutions. Most authors ascribe this observation to hydroxyl ion adsorption at the water-oil interface.

## Surface Complexation Model

In the existing surface complexation models used to describe the electrical properties of the oil-brine interface, the number of sites is always taken as a constant, without actually accounting for the specific compositions of different crude oils^32,33^. The values of the equilibrium constants for the adsorption reactions of ionic species from the brine to the oil surface sites are often obtained from unspecified sources without proper validation. We address these issues by calculating the number of surface sites based on the measured concentrations of the acid and base groups. In addition, we validate the equilibrium constants by using oil in brine zeta potential measurements reported in the literature.

### Methodology

In this work, we consider a diffuse layer surface complexation model to estimate the surface potential, following the approach reported by Brady *et al*.^29^. In practice, the zeta potential is assumed to be equal to the surface potential^13^. Some other works use the Debye Hückel approximation of the Gouy-Chapman theory to link the zeta potential to the surface potential^39,40^. However, this approximation should not be applied when the potentials are high (>25 mV); in such cases the Poisson-Boltzmann equation needs to be solved. For systems containing 1:1 electrolytes, the Poisson-Boltzmann equation has an analytical solution known as the Gouy-Chapman equation^41^.

**Figure 1:**
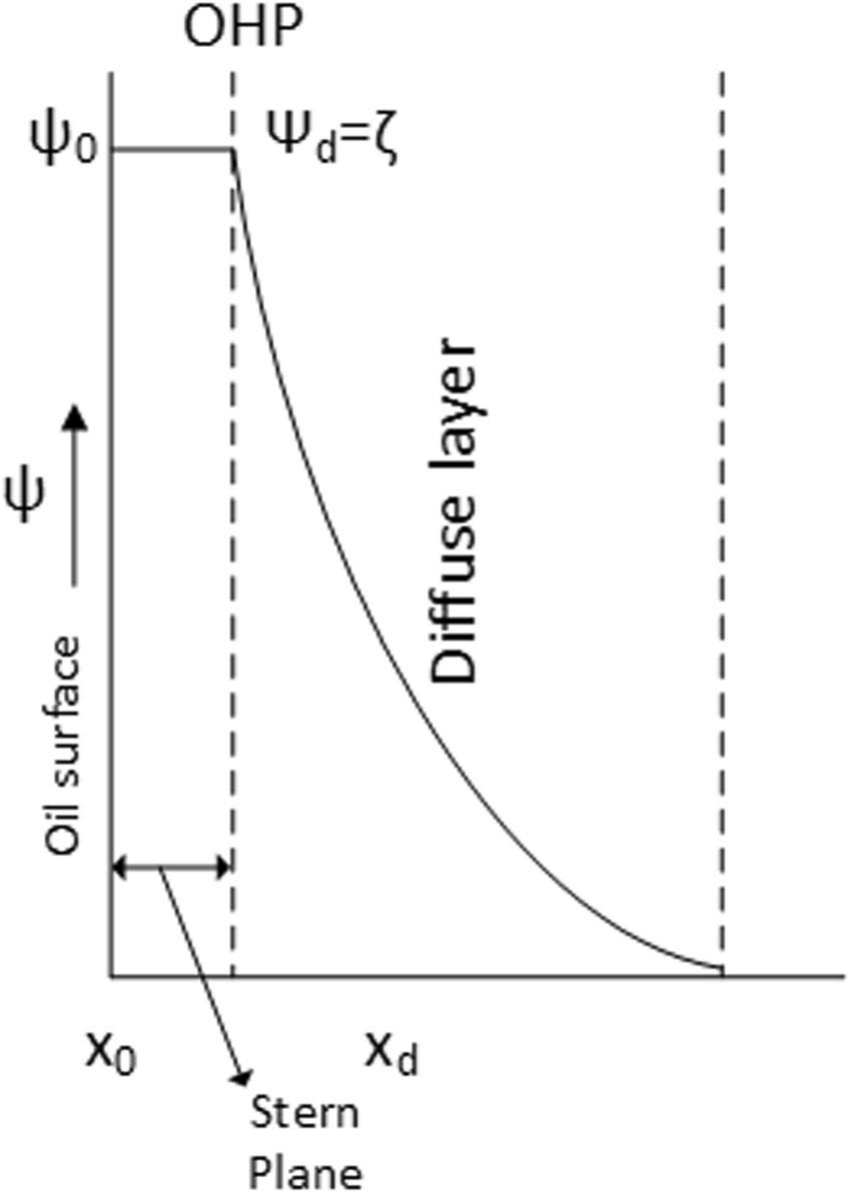
Schematic of the electrical double layer. The zeta potential is assumed to be equal to the potential at the d-plane (OHP).

The electrokinetic measurements rely on the assumption that the zeta potential is the potential at the boundary (shear plane) between the immobile and mobile phases. It is widely accepted that this plane lies close to the outer Helmholtz plane (OHP)^42,43^. Efforts have been made to provide methods to estimate the distance between these planes^44^. Different values for this distance can be found in various publications: 0.33 nm^40^, 1–2 nm^42^, 0.6 nm^25,26^ and 2 nm^45^. Since there is no general agreement on the location of the shear plane, we assume that the zeta potential is equal to the potential at the OHP (ζ = ${\psi }_{d})$ (see Fig. 1), in agreement with many other works, e.g.^46–49^. However, this assumption is not always valid; at high ionic strength the exact location of the slip plane is required for the calculation of zeta potential (see e.g. chapter 1 of^44^).

The zeta potential, ζ, is then calculated in the speciation software PHREEQC^50^ by explicitly defining “diffuse layer” calculations. The potential is computed by explicit integration of the Poisson-Boltzmann equation, following the procedure in Borkovec and Westall^51^ (Eq. 1):

1 $$\frac{{d}^{2}\psi (x)}{d{x}^{2}}=-\,\frac{F}{\varepsilon {\varepsilon }_{0}}\sum _{i=1}^{N}\,{z}_{i}({n}_{i}(x)-{{n}_{i}}^{0}),$$

where the concentration ${n}_{i}(x)\,[\frac{mol}{{m}^{3}}]$ follows a Boltzmann distribution (Eq. 2):

2 $${n}_{i}(x)={{n}_{i}}^{0}\,\exp \,(-\frac{{z}_{i}F\psi (x)}{RT}),$$

where *F* [C/mol] is the Faraday constant, ${\varepsilon }_{0}=8.85\times {10}^{-12}\,F/m$ is the vacuum permittivity, $\varepsilon$ is the relative permittivity of water, *z*_*i*_ is the ionic valency, and ${{n}_{i}}^{0}$ the bulk concentration [mol/m^3^]. For more details on the diffuse layer calculations, the reader is referred to^51^ and^52^.

It should be mentioned that the applicability of the Poisson-Boltzmann equation has limitations at higher ionic strength since it assumes that the ion density profile is only affected by the mean electrostatic potential^53,54^. As explained by Cavalli *et al*.^55^, this equation only provides a good description of the system at low ionic strength where interactions between ions can be disregarded. However, Wang and Chen^56^ showed that Poisson-Boltzmann provides a fairly good estimate of the ion density profile even at high concentrations, e.g., $1M.$

Another model uncertainty is the distribution of species around the oil/brine interface. The surface site density can only be defined when the interfacial region is exactly located^57^. Buckley *et al*.^26^ highlighted that the oil/water interface is negatively charged due to the dissociation of carboxylic acids. However, at lower pH they observed a positive charge, indicating that basic groups are also present at the oil/water interface. Among the acidic and basic functional groups, the naphtenic acids and the pyridinic nitrogen bases are considered the most interfacially active^58^. The naphtenic acids represent a mixture of mainly cyclopentyl and cyclohexyl carboxylic acids with molecular weight between 120–700 and a lower content of other fractions (carboxyphenols, porphyrins, and asphaltene)^59^. On the other hand, other authors consider asphaltene content responsible for the charge development at the oil/brine interface^28,60^. Szymula *et al*.^28^ reported that the surface charge of asphaltenes comes from the dissociation of carboxylic groups and the dissociation and protonation of hydroxyl groups, while Chaverot *et al*.^61^ differentiates between the existence of either acidic (sulfuric or carboxylic) or basic (amine) groups. Generally, most authors accept that the charge at the oil/water interface comes from the ionization of basic and acid surface groups. It is also widely agreed that the isoelectric point is not only affected by the base/acid ratio, but also by the absolute base number, and the base and acid pK values^62^.

The maximum number of acid and basic sites can be calculated from the acid and basic number (Eqs (3)–(4)), respectively, as suggested in the work of Eftekhari *et al*.^39^:

3 $${N}_{S,-COOH}=0.602\cdot {10}^{6}\cdot \frac{TAN}{1000\cdot {a}_{oil}\cdot M{W}_{KOH}}$$

4 $${N}_{S,-NH}=0.602\cdot {10}^{6}\cdot \frac{TBN}{1000\cdot {a}_{oil}\cdot M{W}_{KOH}},$$

where $0.602\cdot {10}^{6}$ denotes the conversion factor from [mol/m^2^] to [#/nm^2^], ${N}_{S,-COOH}$ [#/nm^2^] and ${N}_{S,-NH}$ [#/nm^2^] denote the carboxylic and amine sites respectively, $TAN\,[{\rm{mg}}\,{\rm{KOH}}/{\rm{g}}\,{\rm{oil}}]\,and\,TBN\,[{\rm{mg}}\,{\rm{KOH}}/{\rm{g}}\,{\rm{oil}}]$ denote total acid and basic number respectively, ${a}_{oil}$ [m^2^/g] denotes the specific area of oil, and $M{W}_{KOH}$ represents the molecular weight of potassium hydroxide − 56.1 [g/mol].

The TBN is defined as the mass of KOH (in mg) equivalent to basic components per gram of oil, and the TAN represents the mass of KOH (in mg) required to neutralize acidic components in one gram of oil. For a crude oil with a high acid number (>1 mg/g KOH), most molecules at the oil water interface would be carboxylic acids^63^. Due to their amphiphilicity, acids and bases can adsorb and desorb at the oil-water interface, ultimately reaching a new equilibrium^64^. Generally, the effect of the acid fraction on the oil-water interface has been studied more extensively than the basic fraction. Andersen *et al*.^65^ showed through an infrared spectroscopic analysis of the crude oil/water interfacial film that the concentration of carboxylic acids is higher at the interface. When small amounts of acid were removed from the crude oil, an increased IFT was observed. Through a similar analysis, Guo *et al*.^66^ also proved that active interfacial components, e.g., carboxylic and phenolic groups in the asphaltene fraction, are the main molecules present at the oil/water interface. Rønningsen *et al*.^67^ emphasized the importance of the acid number as indicative of the tendency of crude oil to form stable emulsions with the water. Havre *et al*.^68^ suggested that the amount of different acids in the bulk phase dictates the amount at the oil-water interface and that the dissociated acids are more interfacially active than the undissociated ones. Moradi *et al*.^17^ reported competitive adsorption between asphaltenes and napthenic acids at the oil-water interface. While asphaltenes adsorb at the interface forming a more rigid film structure, the dissociated naphtenic acids can also react with the cations in the brine, forming naphtenate salts. These salts can eventually accumulate at the water-oil interface decreasing the interfacial tension significantly.

The effect of bases on the oil-water interface has been studied less and is still not fully understood^65,69^. The structure of the basic components is mainly derived from pyrrolic and pyridinic groups, with the latter one being the most interfacially active^64,70^. Saliu *et al*.^71^ suggested that the bases affect the oil-water emulsions only by stimulating other active fractions that are present in a latent state in the crude oil. Thus the bases are believed to interact with napthenic acids from the crude oils, leading eventually to emulsification. On the other hand, in interfacial tension studies, Bertheussen *et al*.^64^ observed no interactions between the acids and bases, inferring that they do not exist simultaneously in dissociated form due to similar pK_a_ values. Nenningsland *et al*.^69^ studied the effect of the basic molecules on the water-oil interface and observed changes in the IFT due to the protonation of the bases below pH 5, but no effect was observed on the surface pressure at a liquid/gas interface. However, the decrease in the IFT at low pH (where the protonation of the bases occurs) was less than at high pH (where the dissociation of the carboxylic acids occurs), suggesting that the bases have a lower surface affinity than the naphtenic acid fraction^64,69^. Hutin *et al*.^72^ reported that a higher TAN usually implies a lower IFT; even though basic components are expected to have a similar effect, the transfer of acid groups to the interface is much greater than the transfer of basic species, hence the predominant negative charge of crude oils^73^.

Crude oils with the same basic or acid number may develop a very different surface charge due to a different distribution of surface species. Conflicting views exist on the amount of active material in the bulk oil that is able to travel to the interface. Some authors argue that it is thermodynamically favorable for most bases and acids to accumulate at the oil-water interface rather than staying in the bulk fluid^63^. Others suggest that the total acid/basic/asphaltene content should not be considered as interfacially active, and that the composition at the interface is very different from the composition in the bulk oil or brine^74^. In support of the latter group, Yang *et al*.^75^ consider that only a fraction of the asphaltene content is responsible for emulsion stability. They proposed a method for separating the interfacially active asphaltene fraction, and studied the emulsion stability. The interfacial film formed by this fraction, which represented only 2% of the total asphaltene content, was more rigid than the one generated by the remaining asphaltene fractions. In a later study^76^ they showed that the interfacially active asphaltene fraction has a higher average molecular weight (1000–1200 g/mol) and a higher oxygen content than the remaining asphaltene fraction (700–750 g/mol), associated with sulfoxide groups. Furthermore, Chaverot *et al*.^61^ reported that only 0.015% of the asphaltenes are surface active, and at pH = 2 the concentration of molecules adsorbed at the interface ranged from 1.9 × 10^−7^ to 2 × 10^−6^ mol/m^2^.

In this work, as well as the acid and basic sites considered in the model proposed by Brady *et al*.^29^, an additional type of weak site is included in the model. These weak sites account for the reported adsorption of hydroxyl ions at the non-polar hydrocarbon/brine interface. The performance of the model is assessed with and without these additional sites. While the amine and carboxylic site density (N_s, −NH_ and N_s, −COOH_, respectively) are varied as a function of the AN and BN, the weak site density is constant at 0.3 #/nm^2^, a value that was found to be almost independent of the type of oil^35^. Additionally we restrain the range of oil site densities, based on experimental evidence that shows that only a fraction of the acid and bases will be present at the interface, and that the acidic components are the most interfacially active^65–68^. Thus we correlate the carboxylic sites linearly with the AN, by specifying a minimum N_s,>COOH_ of 0.5/nm^2^ (corresponding to AN = 0.05) and a maximum N_s,>COOH_ of 2.5/nm^2^ (corresponding to AN = 3). Analogously, the amine sites are correlated linearly with the BN, ranging from 0 (BN = 0) to 2 (BN = 3). However, if this approach yields N_s, −COOH_ < N_s, −NH_, we set the amine site density equal to the carboxylic site density, since we found no evidence in the available literature supporting the predominance of basic species over acid species at the brine-oil interface. Furthermore, the maximum value for the acid site density is chosen by analogy with the site density found for oil (benzene and decane) in the presence of surfactants^57^. These upper and lower limits for the AN and BN were used because the available experimental data falls within this range, though AN and BN can show larger values. Thus, in contrast to the Ionizable Surface-Group model used in^25,26^, we do not include the number of sites in the optimization. Rather, we expect to provide a tool that is able to predict the isoelectric point and the zeta potential distribution relative to pH with reasonable accuracy, based on input parameters such as AN and BN. Moreover, if the surface site density is part of the optimization procedure, physically unrealistic values can be obtained, for example values that are higher than the maximum number of sites calculated from the total number of acid and basic molecules. For instance, in^26^ a site density of 2 #/nm^2^ is obtained in the optimization for a North Sea crude oil (ST-86-1) with a low acid number (0.15 mg/g KOH); however, if the site density is calculated from the actual number of molecules and specific surface area (calculated considering the crude oil density and assuming spherical droplets), a value around 0.6 #/nm^2^ would be obtained. For the different experimental datasets considered in this work, the defined surface site densities obtained as a function of the crude oil basic and acid number are shown in Table 1.

**Table 1:** Experimental acid and base number of the crude oils and defined surface site densities in the modeling for the utilized experimental data sets.

Reference	Type of oil	AN (mg/g KOH)	BN (mg/g KOH)	N_−COOH_ (#/nm^2^)	N_−NH_ (#/nm^2^)
Kolltveit^80^	Crude oil A	3	0.8	2.5	0.6
	Crude oil B	2	0.8	1.8	0.6
	Crude oil C	1	0.8	1.15	0.6
Buckley *et al*.^26^	Moutray	0.26	—	0.7	0.1
	Leduc	0.15	—	0.6	0.3
	ST-86-1	0.15	—	0.6	0.03
Chow *et al*.^25^	Moutray	0.26	—	0.7	0.1
	Bitumen	2	—	1.8	0.05
Alshakhs *et al*.^4^	Crude oil	1.15	1.25	1.2	0.8
Nasralla *et al*.^84^	Crude oil A	0.18	1.65	0.6	0.6
	Crude oil B	0.11	0.62	0.3	0.55
Ayirala *et al*.^18^	Crude oil	0.05	0.7	0.5	0.35
Takeya *et al*.^85^	Crude oil	0.39	1.86	0.75	0.75
Lu *et al*.^16^	Oil 1	0.21	5.6	0.6	0.6
	Oil 2	0.18	1.14	0.6	0.6

We also consider additional complexation reactions between the Na^+^ and the carboxylic sites. The reactions included in the model and the initial equilibrium constants are shown in Table 2. These initial intrinsic equilibrium constants are taken from the analogous aqueous reactions (of acetic acid and acetate) from the LLNL (Lawrence Livermore National Laboratory) database^50^. However, the actual occurrence of these adsorption reactions and the distribution of basic and acid sites at the oil surface still needs to be investigated experimentally. The equilibrium constants for reactions 1–3 and 6–7 from Table 2 are optimized (with and without weak sites) by fitting the model to the experimental data from Buckley *et al*.^26^ using a Julia^77^ implementation of the Levenberg-Marquardt optimization algorithm^78,79^. The equilibrium constants for Ca^2+^ are further refined by fitting the model to the experimental data of Chow *et al*.^25^, and the stability constant for the interaction of carboxylic sites with Mg^2+^ ions is considered to be the same as for Ca^2+^. The optimized values are included in Table 2. In comparison with the calcite/brine system, the zeta potential measurements for the oil/brine interface are relatively scarce and are predominantly performed in 1:1 electrolyte systems. Moreover, the model is also optimized when no weak sites and no surface complexation between acid sites and Na^+^ are considered (similar to the model of Brady *et al*.^29^).

**Table 2:** Surface reactions and equilibrium constants before and after optimization.

Surface sites	No	Reactions	log(K)	A	B	C
Amine -NH	1.	-NH + H^+^ → -NH_2_^+^	5.5	7.27	6.70	6.60
Carboxylic -COOH	2.	-COOH → -COO^−^ + H^+^	−4.75	−4.62	−4.65	−4.80
	3.	-COOH + Na^+^ → -COO-Na + H^+^	−4.86	−3.40	−3.67	—
	4.	-COOH + Ca^2+^ → -COO-Ca^+^ + H^+^	−3.82	−3.30	−3.40	−3.4
	5.	-COOH + Mg^2+^ → -COO-Mg^+^ + H^+^	−3.47	−3.30	−3.40	−3.4
Weak -wOH	6.	-wOH→-wO^−^ + H^+^	−8.93	−6.23	—	—
	7.	-wOH + Na^+^ → -wO-Na + H^+^	−8.93	−5.70	—	—
	8.	-wOH + Ca^2+^ → -wO-Ca^+^ + H^+^	−5.85	−4.6	—	—
	9.	-wOH + Mg^2+^ → -wO-Mg^+^ + H^+^	−5.85	−4.6	—	—

Column A refers to the log(k) values obtained when the optimization was performed considering weak sites (Model A); column B gathers the values in the absence of weak sites (model B); column C contains the log(k) values in the absence of weak sites and without considering interaction between the surface of oil and Na^+^ (model C).

## Results and Discussion

**Figure 2:**
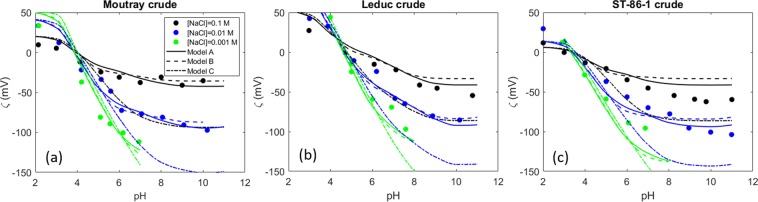
Zeta potential of three different crude oils in three different ionic strength NaCl solutions^26^. The solid lines represent the prediction of the surface complexation model when considering weak sites, the dashed lines correspond to the case which does not consider weak sites (Model B), and the dotted lines represent the fit of the model when no weak sites and no Na^+^ interaction with the crude oil are considered (Model C).

The results of the optimized surface complexation model fitted to the experimental data from Buckley *et al*.^26^ are shown in Fig. 2. Since the base number was not reported in the cited work, we estimated the basic site density from the relationship between acid/basic site density ratio and the isoelectric point (IEP) inferred previously from Kolltveit’s experimental data^80^. We are aware that this approach might be inaccurate, but IEP and BN/AN ratio were previously shown to be correlated^62^. These values are presented in Table 1. The low amine surface site density for the ST-86-1 crude oil is inferred because of the very low IEP (around 3). The solid lines correspond to the fitted model when weak sites and reaction with Na^+^ are considered (Model A), the dashed lines represent the model in the absence of weak sites (Model B), and the dotted lines correspond to the model when no weak sites and no Na^+^ interactions are considered (Model C). It is observed that the model fits the data better it considers the interaction between the sodium and the carboxylic sites, compared to model C that does not consider the Na^+^ reaction. The interaction between this monovalent ion and the carboxylic groups was already investigated and confirmed through molecular dynamic simulations in^81,82^. When this additional reaction was not considered (Model C), the model could not be successfully fitted to the experimental data set. It should be mentioned that the model proposed by Buckley *et al*. fits satisfactorily to their experimental data by considering only de/protonation. However, they used the surface site density as an adjustable parameter, and they also defined a constant slip plane distance of 0.6 nm for the calculation of the zeta potential. Furthermore, since in the diffuse layer model all the ions are assumed to be adsorbed as inner-sphere complexes in the d-plane, the IEP predicted by the models for the Leduc crude (Fig. 2b) changes with the NaCl concentration. However, this experimental data does not suggest changes in IEP with changes in the NaCl concentration, in contrast to the experimental data from Kolltveit^80^.

It can also be observed that model A fits the experimental data slightly better, especially for crude oil ST-86-1 (Fig. 2c). However, the basic site density defined for the ST-86-1 crude is not sufficient to capture the positive zeta potential at lower pH values. Nevertheless, if the basic site density is increased while keeping the same acid (or acid and weak) site density value, the IEP predicted by the model would be shifted to the right. The very low isoelectric point and the more positive zeta potential values might suggest a combination of a greater number of basic species at the surface at low pH with, probably, increased hydroxyl adsorption at the surface, a trend that could be predicted by increasing the number of weak sites. We must note that the acid site density cannot be increased more than the number of acid molecules indicated by the acid number of the oil, even though the acid number measurement is not a sufficient measure of the amount of active species at the interface. All in all, we believe that defining higher surface site densities would not be reasonable since, according to the calculations (applying Eq. 3), there would not be enough acid molecules to yield a higher surface site density, even if all the acid molecules accumulated at the interface. Therefore, it seems plausible that, in this case, the very negative zeta potential at high pH comes from the adsorption of hydroxyl ions at the interface. No major differences are observed between the goodness of fit of Model A and B, since the combination of the equilibrium constants obtained through the optimization yields mostly the same results.

**Figure 3:**
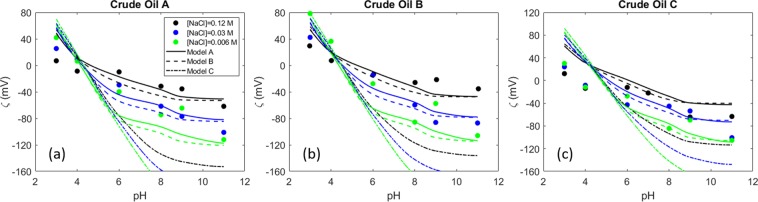
Zeta potential of three different crude oils in three different ionic strength NaCl brines^80^.

Similar experiments were carried out previously by Kolltveit^80^. The prediction of the models for this experimental data set is shown in Fig. 3. Again, the model without Na^+^ reaction (Model C) shows a worse performance than the other two variants. Moreover, the models predict an IEP slightly shifted to the right for Crude Oil A (Fig. 3a) and Crude Oil C (Fig. 3c). Generally, in terms of zeta potential magnitudes, the experimental data is predicted better by Model A, though the performance of Model B is comparable and predicts the IEP slightly better.

Furthermore, although crude oil C has the lowest acid number, (see Table 1) it is observed that at high pH this crude oil shows comparable negative zeta potential values to crude oil A, which has an AN three times higher. The very negative zeta potential values imply that, even though it has a lower acid number, this crude oil might have a higher number of active carboxylic acids at the surface than the other two, yielding the negative charge at high pH through the dissociation of the acids. Similarly, even though all crude oils have the same basic number, crude oil B shows considerably higher positive zeta potentials at low pH, especially at the lowest ionic strength. This indicates that the basic and acid number are not reliable indicators of the amount of active molecules at the oil surface. This phenomenon complicates the development of consistent models for the oil surface charge prediction, since there is no strong premise to back the surface site density definition.

**Figure 4:**
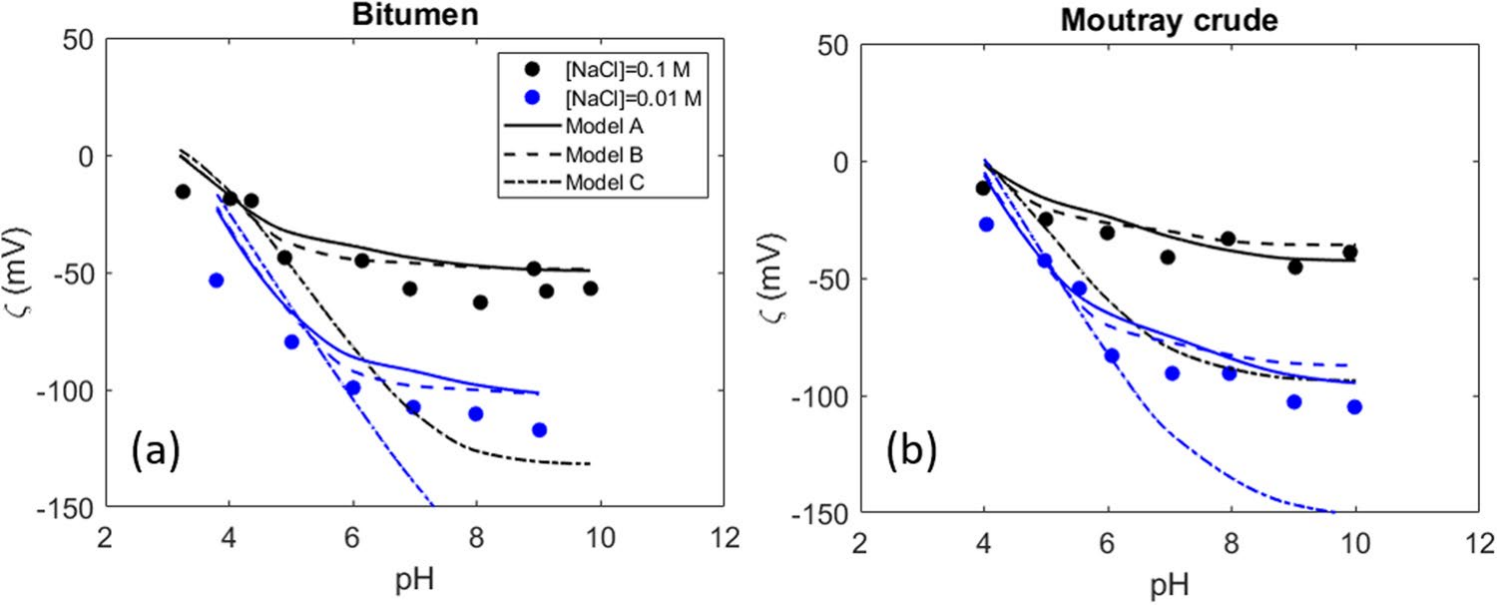
Zeta potential of (**a**) bitumen and (**b**) crude oil sample in two different ionic strength NaCl brines^25^.

Chow *et al*.^25^ performed zeta potential measurements of bitumen and crude oil sample in NaCl brine. The acid number and the defined surface site densities of the samples are shown in Table 1. In this case, the base number was not reported. Therefore, it is estimated using the same procedure as for the experimental data of Buckley *et al*.^26^. The zeta potential prediction at two different ionic strength NaCl solutions is shown in Fig. 4. Although the model accurately replicates the zeta potential trend, the experimental values are slightly lower than the model predictions. Model A provides a better prediction for the Moutray crude oil than model B. The prediction of model C deviates considerably at higher pHs, especially at lower ionic strength.

**Figure 5:**
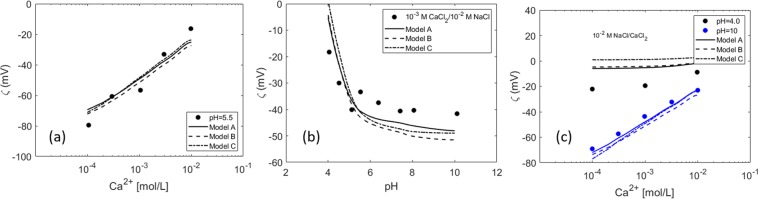
Zeta potential measurements^25^ of (**a**) bitumen at increasing Ca^2+^ concentration at a fixed pH = 5.5; (**b**) crude oil sample in a CaCl_2_/NaCl brine mixture at different pHs (**c**) crude oil in a brine mixture with increasing CaCl_2_ concentration at two different pH values. The equilibrium constant for the Ca^2+^ adsorption reaction was obtained by fitting the model to this experimental data.

Chow *et al*. also studied the effect of calcium ions by measuring the zeta potential in NaCl/CaCl_2_ brine mixtures. The results of the model for this experimental data are shown in Fig. 5. The model correctly predicts the decrease in the negative magnitude of the zeta potential with increasing Ca^2+^ concentration (Fig. 5a) and the fast decrease in the zeta potential with pH. At first, a higher number of carboxylic sites are available to form complexes with the Na^+^ and Ca^2+^ in the brine, increasing the surface charge. As soon as the surface sites are occupied, the zeta potential does not change further (Fig. 5b). The model satisfactorily predicts the diminished calcium effect observed at pH = 4 due to reduced complexation between carboxylic acids and cations at lower pH; compare the gradient of zeta potential increase with increasing Ca^2+^ concentration at pH = 4 and pH = 5.5 with that at pH = 10 (Fig. 5c). A similar fit is obtained when weak sites, no weak sites or no Na^+^ are considered, with a slightly better performance of the model that considers weak sites (model A).

**Figure 6:**
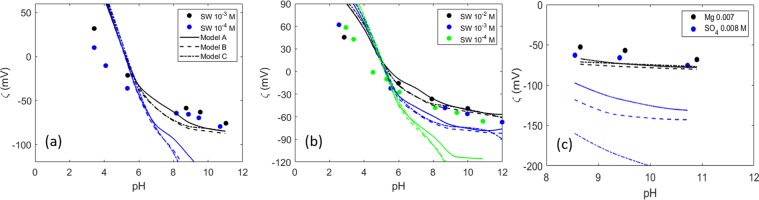
Zeta potential of crude oil in different brine compositions and different ionic strength^4^.

Alshakhs *et al*.^4^ measured the zeta potential of crude oil in different brine mixtures. They conducted the measurements at even lower ionic strength and also added Mg^2+^ and SO_4_^2−^ to the brine. The results of the model are compared with this experimental data set in Fig. 6.

The SW brine (Fig. 6a) is a combination of Na^+^, Mg^2+^, SO_4_^2−^ and Cl^−^ ions. The MgSO_4_ brine (Fig. 6b) has the same ionic composition but enriched in Mg^2+^ and SO_4_^2−^. Lastly, the Mg brine contains no SO_4_^2−^ and the SO_4_ brine contains no Mg^2+^ (Fig. 6c). The AN and BN of the crude oil are shown in Table 1. This crude oil had a significantly higher acid number than the samples studied by Chow *et al*.^25^ and Buckley *et al*.^26^; but, surprisingly, the zeta potential values are less negative at high pH, even when measured at lower ionic strength. This could be a consequence of the Mg^2+^ which forms positive complexes with the carboxylic sites and makes the zeta potential less negative. Moreover, this experimental data shows almost no variations in the zeta potential with the ionic strength, in contrast to the other experimental data sets used in the present work. The measurements suggest that sulfate has a similar effect on the oil/brine zeta potential as Mg^2+^ (Fig. 6c), i.e., that of increasing the zeta potential. In contrast, other works suggest that sulfate ions interact only with the mineral and not with the crude oil^29^. A higher contact angle was observed by Alshaks and Kovscek^4^ in the presence of sulfate ions, on which basis they suggested that sulfate does not increase the water wetness and that magnesium ions are more effective in altering the wettability toward stronger water wetness. Our models predict larger differences between the zeta potential at the three different ionic strengths than are observed in this experimental data set. Moreover the base number reported for this crude oil is higher than the acid number, and, due to the combination of defined surface site densities, our models predict a higher isoelectric point, suggesting that the actual amine site density could be lower than that specified. However, differences between the model and the experimental data could also arise because of incorrect equilibrium constants. In the model, all the acid and basic sites are considered to be identical; in reality, the AN and BN include acids and bases of different types which could certainly react differently with the ions in the brine.

**Figure 7:**
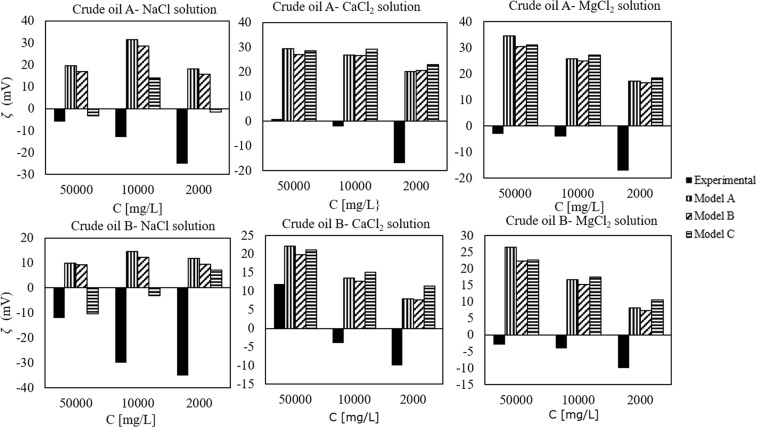
Zeta potential of two different crude oils in NaCl, CaCl_2_ and MgCl_2_ aqueous solutions at a pH ≈4. Data from^84^.

Nasralla *et al*.^83^ investigated the role of double layer expansion in the improved oil recovery during modified salinity water flooding by measuring the zeta potential at both mineral/brine and brine/oil interfaces. Among multiple mechanisms for this recovery process, they considered the expansion of the double layer as the most dominant mechanism. They did not observe any increase in the oil recovery in tertiary mode (i.e., injecting low salinity brine into a core that is already flooded with the initial formation brine). This emphasizes the importance of the initial wetting state condition of the mineral in the success of smart water flooding. To explain these observations, a later work studied the effect of the injection water salinity and cation type on the zeta potential^84^. They measured the zeta potential of two different crude oils in NaCl, CaCl_2_ and MgCl_2_ solutions at a pH value around 4. The AN and BN of the oil samples are shown in Table 1. The results of our model are shown in Fig. 7. These measurements are performed at a higher ionic strength than the previous data sets. This experimental data also shows that Mg^2+^ and Ca^2+^ mostly interact in the same way with the crude oil acid sites. This justifies the use of the same equilibrium constant for the adsorption of Ca^2+^ and Mg^2+^ on the carboxylic sites. It also questions the accuracy of the models that include the interaction of Mg^2+^ with the calcite surface sites but not with the oil^30^. Both crude oils have a low acid number and higher basic number. Even though crude oil B has a lower AN, the experimental zeta potential values are more negative in the NaCl brine, while less negative values are obtained experimentally in the presence of CaCl_2_ and MgCl_2_. Our model cannot capture this trend because the surface site density is defined only by the AN and BN. It is possible to match the experimental data by including more types of surface sites and optimizing the equilibrium constants. However, the number of variables in the optimization becomes more than the number of data points, which makes the model overly complicated and impractical.

**Figure 8:**
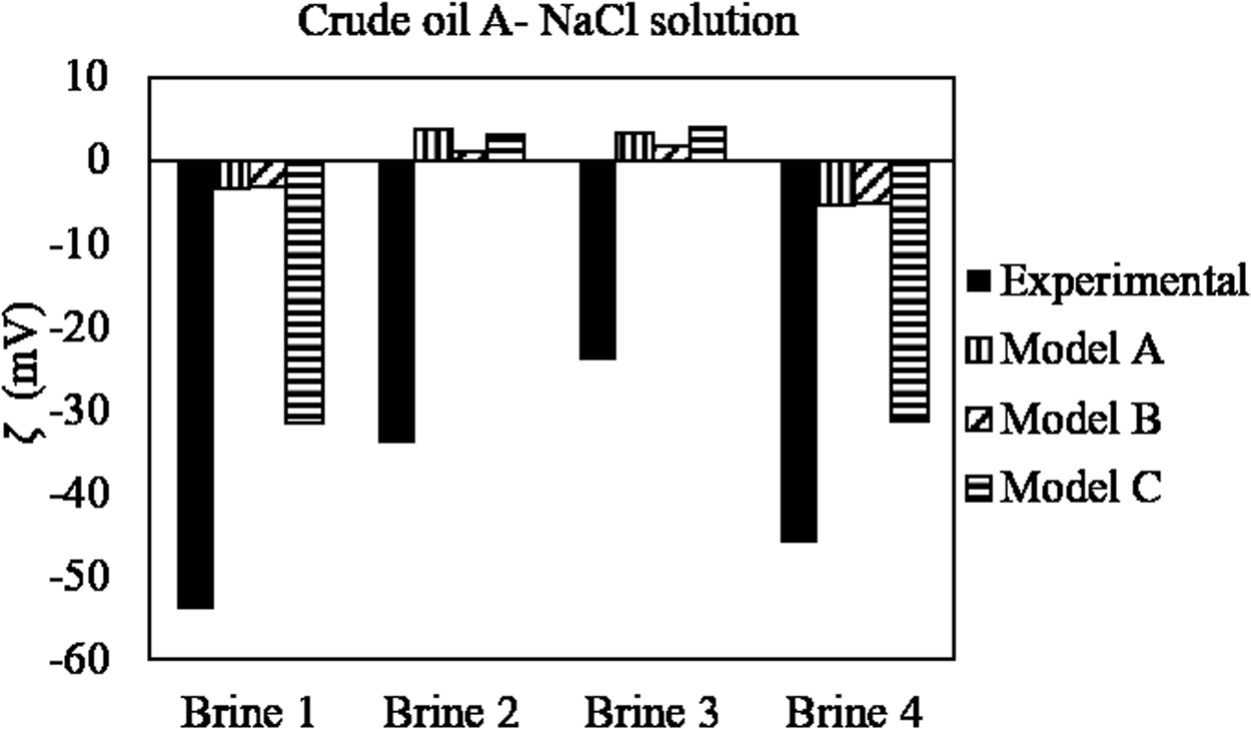
Zeta potential of crude oil in four different brine compositions^18^. All the measurements were performed at a pH around 6. Composition of the brines: brine 1- Na^+^ and Cl^−^; brine 2- Mg^2+^ and Cl^−^; brine 3- Ca^2+^and Cl^−^; brine 4: Na^+^ and SO_4_^2−^. All the brines have similar ionic strength.

Overall, the predictions of the model are not satisfactory in these experiments, since in most cases the model does not even predict the correct polarity of the zeta potential. This indicates that the number of amine sites defined in the input to the model must be lowered in order for the model to be able to capture the IEP, which from these measurements appears to be somewhat lower than 4. The discrepancy between the model and the experimental evidence could be a consequence of the higher ionic strength of the brines in these experiments; note that the optimization of the model parameters was performed using zeta potentials obtained at lower ionic strength. Moreover, in this work a high oil/brine volume ratio is used in the electrophoretic measurements (20%), whereas much lower values (<1%) were reported in other works^18,25,26,36^. The modeling of these data would probably benefit from more information on the experimental conditions (i.e., oil drop size, closed or open system, etc.). Ayirala *et al*.^18^ performed zeta potential measurements on a crude oil with very low AN and high BN (Table 1) in four different brines. Theoretically, a positive surface charge would be expected throughout the whole pH interval, since the fraction of acids undergoing dissociation at higher pH is expected to be very low. As observed in Fig. 8, if only acid and basic sites are considered, the predicted zeta potential has a lower magnitude than is measured. In this case, model C provides the best prediction for the systems that contain NaCl and Na_2_SO_4_. This shows that including Na^+^ surface complexation reaction or defining weak sites improves the goodness of the fit. The fact that the pH of the oil-brine emulsions is not reported before the electrophoretic measurements (the pH of the brines is reported instead) can also lead to differences between the predicted and the experimental zeta potential values. The authors mention that the negative surface charge of the oil can be explained by the brine pH of around 6. However, knowing the actual pH of the oil-brine system would facilitate the comparison between the results from the model and the experimental data.

**Figure 9:**
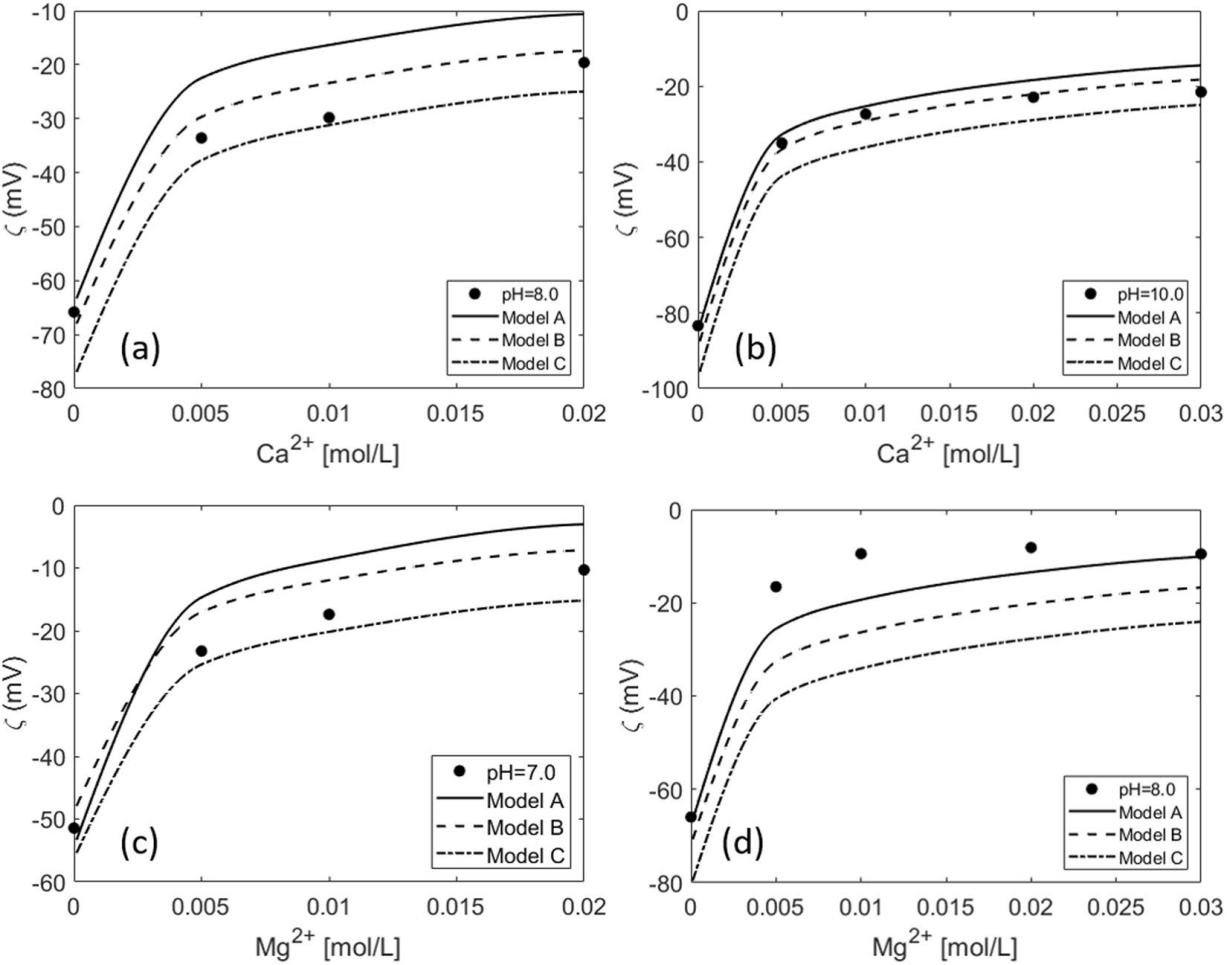
Experimental data from^85^: Measurements at varying (**a**) CaCl_2_ concentration at a fixed pH = 8 (**b**) CaCl_2_ concentration at a fixed pH = 10 (**c**) MgCl_2_ at a fixed pH = 7 (**d**) MgCl_2_ at a fixed pH = 8.

Takeya *et al*.^85^ measured the oil/brine zeta potential in electrolyte solutions containing Na^+^, Cl^−^, Ca^2+^, and Mg^2+^. They performed these experiments at 50 °C and only at pH values above 7. Based on these measurements they proposed a CD-MUSIC model that considers the alterations in the surface charge that are a result of the deprotonation of carboxylic acid sites and their complexation with divalent cations in the solution. Since no basic sites are considered, their model would not be able to capture the positive zeta potentials at low pHs. Figure 9 shows the experimental data at increasing calcium or magnesium concentration at a fixed pH. The three models correctly predict the shift in the zeta potential towards more positive values with increasing divalent cation concentrations. The model that does not consider weak sites (Model C) predicts more negative values since there are fewer sites available for interaction with Ca^2+^ and Mg^2+^.

**Figure 10:**
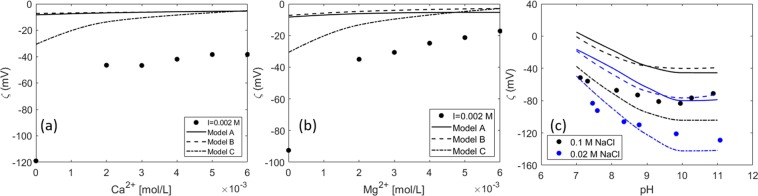
Experimental data from^85^: Measurements at a constant ionic strength (fixed with NaCl solution) at increasing: (**a**) CaCl_2_ concentration (**b**) MgCl_2_ concentration (**c**) 0.1 and 0.02 M varying pH.

Figure 10 shows zeta potential measurements at a constant ionic strength of 0.02 M with increasing Ca^2+^ (Fig. 10a) and Mg^2+^ (Fig. 10b) concentrations. No pH values are reported for these measurements, and so they were modeled by assuming the equilibrium pH predicted by PHREEQC. As observed in Fig. 10a,b, the model does not predict the jump in the zeta potential between 0–0.002 M Ca^2+^. More measurements would be needed at intermediate Ca^2+^ values to see the actual trend of the data between these two points. Moreover, comparing Fig. 10a and the changes in the zeta potential with pH at a constant ionic strength (Fig. 10c) shows that the value of 120 mV in 0.02 M NaCl corresponds to approximately a pH = 10. This means that the first measurement in Fig. 10a would correspond to a very high pH (≈10). However, the equilibrium pH predicted by PHREEQC for that system is around 6.6, which could explain the differences in the prediction of the model and the experimental zeta potential. This demonstrates the importance of pH monitoring during the zeta potential measurements. Having the pH as an input to the model would add more consistency to the comparison between the model and the experimental data. Finally, differences in the predicted and measured values may also arise due to the higher temperature of the oil-brine system. In this study, the standard enthalpy of the surface complexation reactions (see Table 2) were taken from the LLNL database for the analog aqueous phase reactions, and the equilibrium constants were then calculated at the specified temperature from Van’t Hoff equation within PHREEQC.

**Figure 11:**
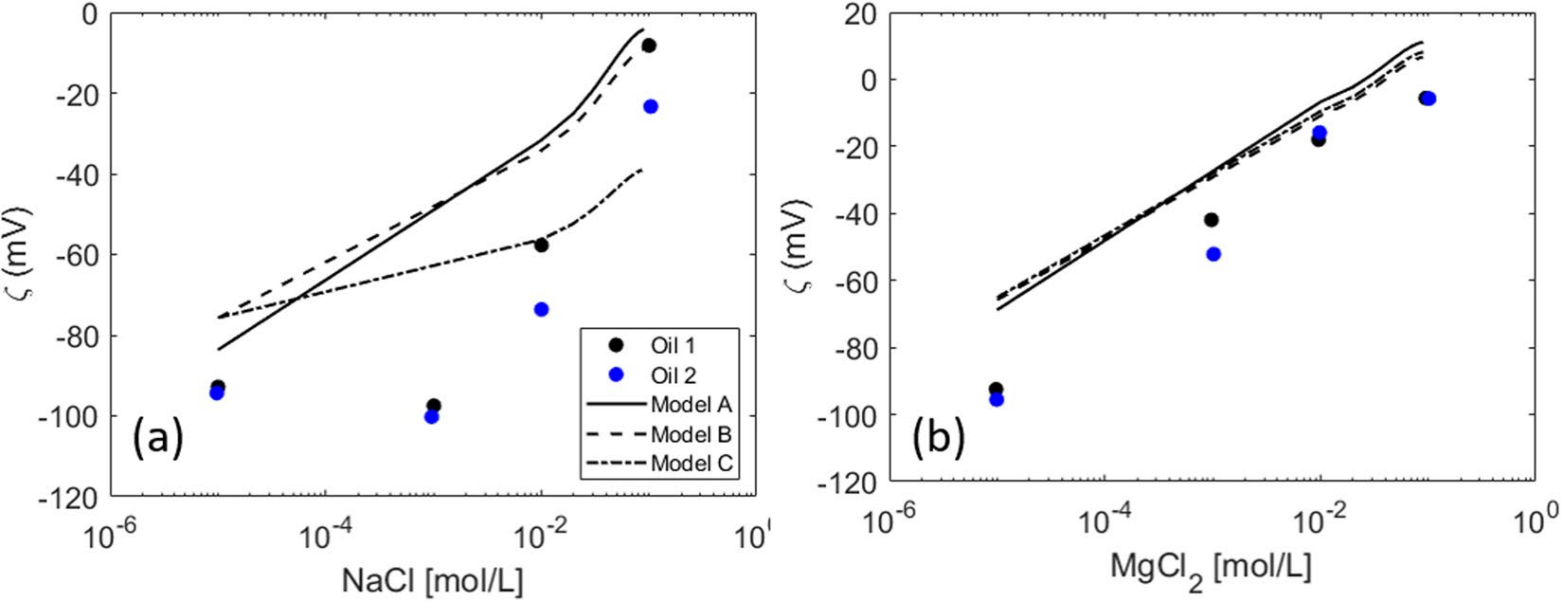
Zeta potential measurements for two different crude oils^16^ with increasing (**a**) NaCl concentration (**b**) MgCl_2_ concentration.

Lu *et al*.^16^ studied the temperature effect on the interactions between the calcite-brine and oil-brine interfaces. They measured the zeta potential of the brine oil interface for two different types of oil in NaCl and MgCl_2_ from low (10^−5^ M) to very high (3 M) concentrations. Only the experimental data up to 0.1 M is modeled here. Moreover, following the procedure used to link the surface sites to the TAN and TBN, we used the same amine and carboxylic sites for both crude oil samples. This is also in fair agreement with the experimental data that shows no major differences in the zeta potential measurements for the two crude oil types. Since no information on the equilibrium pH is provided, the data is modeled assuming the equilibrium pH predicted by PHREEQC. However, as explained earlier, this might be very different to the actual pH of the system, resulting in discrepancies between the predicted and measured zeta potential. Generally, the model prediction for the NaCl system (Fig. 11a) is not as good as for the system with Mg^2+^ (Fig. 11b). A nonmonotonic behavior of the zeta potential is observed experimentally in the presence of monovalent electrolytes, which cannot be captured by the models if the equilibrium pH is assumed. Specifying the measured pH would make the predictions of the model and the interpretation of the experimental data more reliable.

**Figure 12:**
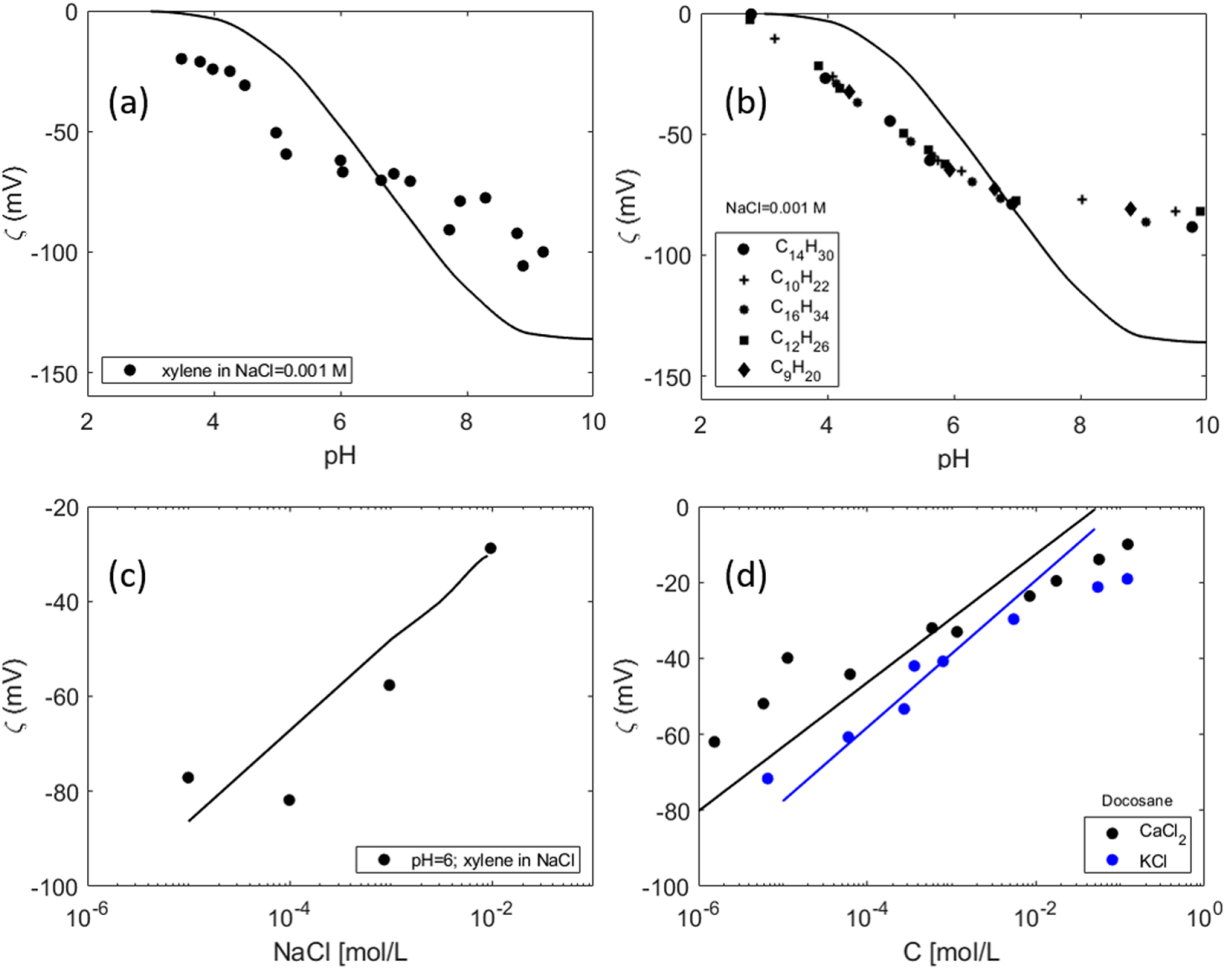
Prediction of the model with weak sites for (**a**) experimental data from^36^: zeta potential measurements of xylene in NaCl solution [0.001 M] at different pH; (**b**) experimental data from^37^-zeta potential measurements with pH of different alkanes in [0.001 M NaCl solution] (**c**) experimental data from^36^- zeta potential measurements of xylene in different NaCl concentrations at a fixed pH = 6 (**d**) experimental data from^38^- zeta potential measurements of docosane in CaCl_2_/KCl solutions; no pH measurements are reported, and the predicted zeta potential is at the equilibrium pH predicted by PHREEQC.

Lastly, model A is tested against zeta potential measurements of non-polar hydrocarbons (with no ionizable components) in aqueous solutions (Fig. 12).

The predicted zeta potential follows the general trend of the experimental measurements. It is observed that the values reported in^36^ (Fig. 12a) are higher than the ones in^37^ (Fig. 12b) at similar experimental conditions. Therefore, the accumulation of hydroxyls at the surface might not be the only mechanism responsible for the surface charge generated at the non-polar hydrocarbon-water interface. The physical properties of each non-polar oil, and possibly the presence of impurities, might also lead to differences in the surface charge. Differences in the measured zeta potential could also arise due to variations in the oil specific area (different oil drop sizes in the preparation of the emulsions) or from a different oil/water volumetric fraction used in the experiments (0.05% in^36^ and 0.5% in^37^). The uncertainty in predicting the zeta potential is also made obvious in the work of Marinova *et al*.^36^, where it was shown that the error associated to the measurements in Fig. 8a reaches ±15 mV. Additionally, the equation used to relate electrophoretic mobility and zeta potential can also lead to different estimations of the electrokinetic potential. While Smoluchowski’s equation was used in^36^ to estimate the zeta potential from the electrophoretic mobility, no information on this aspect is provided in^37^. Moreover the agreement between the model and the experimental data in Fig. 12d is fairly good, considering that no information on the pH was reported and that the calculation is based on the equilibrium pH predicted by PHREEQC, which, as discussed before, is probably different to the pH in the real system.

## Conclusions

In this work, we propose a diffuse layer surface complexation model to predict the zeta potential at the oil-aqueous solution interface, assuming that the presence of carboxylic and amine sites at the oil surface is linearly dependent on the TAN and TBN. A third type of weak site is included to account for the reported adsorption of hydroxyls at the interface. This model is a useful tool to determine the changes in the wettability, assess the optimum water composition during low salinity water flooding, and provide insight into emulsion stability. The key findings extracted from this study are summarized as follows:

- The success of the model’s predictions relies heavily on the definition of active sites at the oil surface. At the moment, the acid and basic number are the main parameters used to estimate the amount of surface active material in the crude oil. However, these do not give an exact indication of the extent of active species that actually ‘travel’ to the interface. Therefore, the AN and BN do not display a clear picture of the type and distribution of reactive sites at the oil surface.
- The addition of a complexation reaction between the carboxylic sites and the Na^+^ was necessary to provide a satisfactory zeta potential prediction.
- Including weak surface sites improved the prediction of the model, especially for crude oils with very low AN, which still show a very negative zeta potential. Under these circumstances, the negative zeta potential is probably a consequence of the adsorption of hydroxyl ions at the interface, which is described by the addition of weak surface sites.
- The performance of the model was generally satisfactory at low ionic strength (up to 0.1 M) while higher deviations were observed at higher ionic strength (1.5 M). The lack of consistent experimental evidence and the inherent limitations of the Poisson-Boltzmann equation at high salinity increased the discrepancy between the model and the measured zeta potential.
- Generally, the model performed worse at reproducing experimental data sets that do not report the pH. From the analyzed experimental data, it could be inferred that the pH of the oil-brine system predicted by PHREEQC is different from the one in the experiments. More insight can be obtained from the modeling of the data if the experimental conditions are properly described.
